# Practical Notes on Cutaneous Subjects

**Published:** 1874-10-01

**Authors:** Tilbury Fox

**Affiliations:** M.D. Lond., F.R.C.P.


### PRACTICAL NOTES ON CUTANEOUS SUBJECTS.

By Tilbury Fox, M.D., Lond., F.R.C.P.

*From the London Lancet, Sept.*, 1874.

### SYPHILITIC TUBERCLES ABOUT THE NOSE.

I HAVE seen strange mistakes made in regard to the nature of specific indurations or tubercular formations about the nose. The occurrence of syphilitic inflammation or infiltrations in circumscribed spots about the nose, constituting the sole syphilitic cutaneous manifestation present, is not uncommon. The favorite seat of these infiltrations is the hollow formed by the junction of the ala of the nose with the cheek. These formations may be as small as, and even smaller than, a split pea, or as large as an almond or larger. There may be one or several. Generally speaking there are one or two packed together. In some cases the nose about its tip or side, or in both situations, appears enlarged from excessive tissue ; whilst it is at the same time redder and hotter than usual, and distinctly indurated to the touch.

More rarely the general enlargement of the nose is excessive. The tubercles, or the more general enlargement, may be the seat of ulceration or crusting. No such condition as the last described could be the result of simple inflammation. The syphilitic disease begins and progresses slowly and indolently; and the enlargement is due to the formation of a fleshy-like mass, firm and reddish—a neoplasm in fact. The neoplasm in the form of a tubercle or a more diffused infiltration is firm, not very vascular; it *tends* to crust freely and to ulcerate—characters that bespeak its syphilitic nature. The lupus neoplasm is softer, more vascular, gelatinous-looking, and, if small, does not crust, except in strumous subjects, but is covered by thin scales, which are closely adherent; and, further, the tubercles are not multiform, save but very rarely. But the diagnosis is further set at rest by the discovering of some concomitant syphilitic lesion of the throat or the tongue, by the presence of nocturnal bony pains, &c., although there may be no additional evidence of skin syphilis.

These syphilitic tubercles are often taken to be acne indurata spots; but they have none of the characters of an inflamed sebaceous gland, and especially no central aperture indicative of the follicular opening; they are primarily solid (new) formations, and they tend to ulcerate and leave pits behind on their disappearance, unlike ordinary acne spots. I have seen many cases of these syphilitic tubercles or slight infiltrations about the nose which have been treated for a long time without any benefit because their nature was not recognized; but if the fact of the disease beginning as a new formation be attended to, the observer will be at once put upon his guard against error, and many a patient will be saved from ugly ulceration and deformity of the ala of his nose by timely treatment.

### UNCONSCIOUS SCRATCHING.

I am convinced that sufficient attention is not paid to the evil effects of scratching upon skin diseases accompanied by pruritus, and especially by such as may be termed unconscious scratching—i. e., that which is practiced during the night. To the case of old people suffering from pruriginous affections these remarks particularly apply. I was recently consulted by a gentleman in his fifth-eighth year, who had been greatly distressed and worried by an attack of cutaneous pruritus, for which he had failed to get relief, and which he aggravated greatly by savagely excoriating the skin with the nails, thereby inducing a crop of what is known as pruriginous papules (inflamed follicles and papillæ in a state of excoriation). The cause of his malady was overwork and want of fresh air no doubt; this, however is not the point I want to notice, but the effect of scratching during sleep practiced perfectly unconsciously upon himself. He told me that his mornings were miserable on account of the burning and soreness he felt in his skin. He imagined that he had exacerbations of his disease in the mornings; but I observed that each morning the skin appeared to be more than usually excoriated, and on requesting his wife to watch his doings at night, it appeared that he was in the habit of almost continuously scratching himself in various parts of the body when in sound sleep. He had no idea that he habitually so scratched his skin; but attributed the ill effects of the scratching to an aggravation, natural as it were, of the disease. The disease in his case was speedily cured, though it had lasted some time, by anointing the body with simple oil, and tying the hands in gloves, so that the nails could inflict no injury upon the skin when he was not watched in the night. The case illustrates the importance of attending to "little matters" in the treatment of skin diseases, and especially in regard to scratching.

### SYPHILITIC PEMPHIGUS IN AN ADULT.

As instances of syphilitic pemphigus are "few and far between" in private practice, the narration of the following case, which came under my observation in March, 1873, will be followed with interest.

The patient was a gentleman aged twenty-five, sent to me by his medical man from the country. He informed me that he had "had a great deal of worry and trouble of late," and had suffered much from "migrain." The disease for which I was immediately consulted attacked him first in October, 1870, about the hands and feet, and the interior of the mouth was so badly affected that he "could not eat anything," and the patient added that "the throat was awfully bad." He had been attacked by seven outbreaks in all, including the one I saw. The outbreaks kept him in bed a fortnight, and left him in a very weak and depressed condition. When any outbreak was severe, the patient suffered from intense headache during its height for about twenty-four hours. He had been subject to neuralgic pains, and occasionally to rheumatic pains. He had been very low-spirited. The body had never been attacked by the eruption; the penis, however, had suffered of late.

The last attack began on March 6th by a little speck on the centre of each hand, the patient being very low and weak. The lip became sore on its outside and "broken." On March 8th another spot appeared on the thumb. On the 11th other spots had appeared about the fingers, and the first which came had assumed the aspect of little bullæ; the gums had also become tender, and the spots on the 14th had reached the size of a large split-pea. On the 15th the patient felt so weak that he had to go to bed; the throat got very sore and the bullæ filled with bloody fluid and looked like black grapes. The feet now became affected. On March 20th the patient was still in bed; headache came on and lasted all day, and the man suffered from great pain in the limbs and febrile disturbance. He got up on the 24th of March (eighteenth day of disease) for the first time since he took to bed. When I saw the patient on March 28th—that is, the twenty-second day of the attack, there were about sixteen bullæ on each hand, of various sizes and different stages of evolution, some being much indurated at their bases and some freely coated over; and about ten on each foot. Patient's tongue exhibited white patches and fissures on the sides, and there was also evidence of syphilitic ulceration about the throat on each side. His wife was confined of her first child in February, five weeks before her expected time, and the child only lived two days.

*Remarks.*—I base my diagnosis of syphilis upon the general state of the patient, which showed that he was to some extent cachectic; upon the frequency of the attacks of headache; the occurrence of rheumatic and neuralgic pains, especially about the head; the evidence of syphilitic mischief in the throat and about the tongue; the seat of the bullous eruption—viz., the soles of the feet and the palms of the hands; the indolent character of the eruption—viz., the slow development of the bullæ, their tardy progress, the presence of sanious contents, and particularly the induration left behind by them; upon the premature confinement of his wife with what was practically a still-born child; and, lastly, the effect of anti-syphilitic treatment in relieving the patient.

---

SURGICAL ANÆSTHESIA.—M. Fornes, a French naval Surgeon, urges the advantage of putting a patient asleep by administering chloral hydrate previously to his inhaling chloroform for the purpose of anæsthesia.—*Le Movement Medical*, June 27, 1874.

A NEW DIAGNOSTIC SIGN OF AMYLOID DEGENERATION.—Dr. Lionville, of Paris, has observed the presence in the urine of epithelial cells having undergone amyloid degeneration in the adult. He advises therefore in all cases where amyloid degeneration is suspected to exist—namely, those in which chronic diarrhœa, with cachectic symptoms and tumefaction of the spleen, are observed—to search in the urine for this additional sign.

